# Human tumour-associated cell adhesion protein MN/CA IX: identification of M75 epitope and of the region mediating cell adhesion

**DOI:** 10.1054/bjoc.2000.1111

**Published:** 2000-06

**Authors:** J Závada, Z Závadová, J Pastorek, Z Biesová, J Jez̆ek, J Velek

**Affiliations:** Institute of Molecular Genetics, Academy of Sciences of the Czech Republic, Flemingovo nam. 2, Prague, 16637, Czech Republic; Institute of Virology, Slovak Academy of Sciences, Bratislava, 84246, Slovakia; Institute of Organic Chemistry and Biochemistry, Academy of Sciences of the Czech Republic, Prague, 16610

**Keywords:** cell adhesion molecules, carbonic anhydrase, tumour immunology, phage display, drug design

## Abstract

MN/CA IX is a cell surface protein, strongly associated with several types of human carcinomas. It exerts activity of carbonic anhydrase and capacity of binding to cell surface receptors. In the present work, we used affinity purified MN/CA IX protein to demonstrate that the cells adhere to immobilized MN/CA IX and that the monoclonal antibody M75 abrogates cell attachment to MN/CA IX. Using synthetic oligopeptides, we identified M75 epitope and located it in the proteoglycan domain, which contains a sixfold tandem repeat of six amino acids GEEDLP. From phage display library of random heptapeptides we identified and chemically synthesized those which compete for the epitope with M75 and inhibit adhesion of cells to MN/CA IX. These heptapeptides might serve as lead compounds for drug design. © 2000 Cancer Research Campaign


British Journal of Cancer (2000) 82(11), 1808–1813
© 2000 Cancer Research Campaign
DOI: 10.1054/ bjoc.2000.1111, available online at http://www.idealibrary.com on IDEAL®

### Human tumour-associated cell adhesion protein MN/CA IX: identification of M75 epitope and of the region mediating cell adhesion

**J Závada[1], Z Závadová[1], J Pastorek[2], Z Biesová[2], J Ježek[3] and J Velek[3]**

[1]Institute of Molecular Genetics, Academy of Sciences of the Czech Republic, Flemingovo nam. 2, 16637 Prague, Czech Republic; [2]Institute of Virology, Slovak Academy of Sciences, 84246 Bratislava, Slovakia; [3]Institute of Organic Chemistry and Biochemistry, Academy of Sciences of the Czech Republic, 16610 Prague

**Summary** MN/CA IX is a cell surface protein, strongly associated with several types of human carcinomas. It exerts activity of carbonic anhydrase and capacity of binding to cell surface receptors. In the present work, we used affinity purified MN/CA IX protein to demonstrate that the cells adhere to immobilized MN/CA IX and that the monoclonal antibody M75 abrogates cell attachment to MN/CA IX. Using synthetic oligopeptides, we identified M75 epitope and located it in the proteoglycan domain, which contains a sixfold tandem repeat of six amino acids GEEDLP. From phage display library of random heptapeptides we identified and chemically synthesized those which compete for the epitope with M75 and inhibit adhesion of cells to MN/CA IX. These heptapeptides might serve as lead compounds for drug design. © 2000 Cancer Research Campaign

**Keywords:** cell adhesion molecules; carbonic anhydrase; tumour immunology; phage display; drug design

MN/CA IX protein (initially termed MN) was discovered in HeLa cells using monoclonal antibody (mAb) M75 (Pastorekova et al, 1992). Its possible role in oncogenesis was suggested by two facts: expression of MN/CA IX correlated with tumorigenicity of HeLa × fibroblast hybrids; it was detected in clinical specimens of cervical and certain other carcinomas, but it was absent in normal tissues of corresponding organs (Závada et al, 1993). *MN/CA9* cDNA (Pastorek et al, 1994) and gene (Opavsky et al, 1996) have been cloned; predicted protein consists of signal peptide (SP), proteoglycan-related sequence (PG), carbonic anhydrase domain (CA), transmembrane segment (TM) and short intracellular tail (IC). MN/CA IX protein is a cell adhesion molecule; its CA is enzymatically active (Zavada et al, 1997). MN/CA IX protein is almost 100% associated with cervical carcinomas (Liao et al, 1994), with carcinomas of oesophagus (Turner et al, 1997) and with renal clear cell carcinomas (Liao et al, 1997). It was also detected in a high percentage of colorectal (Saarnio et al, 1998*a*) and lung (Vermylen et al, 1999) carcinomas. Therefore, MN/CA IX is a promising biomarker in tumour diagnostics. Characteristically, MN/CA IX in human carcinomas is expressed ectopically; normally it is present in stomach and bile duct mucosa (Liao et al, 1994) and in rapidly proliferating normal cells in small intestine (Saarnio et al, 1998b). An antigen specific for renal cell carcinomas was discovered using mAb G250 (Oosterwijk et al, 1986) and was found to be identical with MN/CA IX (Uemura et al, 1997). This antigen appears to be an excellent target for immunotherapy (Steffens et al, 1997; Uemura et al, 1997). Until now, molecular basis of involvement of MN/CA IX in carcinogenesis has remained unclear. One of the possible leads could be the fact that MN/CA IX is an adhesion molecule presumably engaged in cell-to-cell communication (Závada et al, 1997). In this paper we undertook the first steps towards elucidation of the role of MN/CA IX protein in communication among the cells. We attempted to identify its binding site for cell surface receptors. This was facilitated by the finding that mAb M75 blocks adhesion of cells to affinity-purified MN/CA IX protein.

*Received 16 September 1999*
*Accepted 27 January 2000*

*Correspondence to:* J Závada

### MATERIALS AND METHODS

### Affinity chromatography of MN/CA IX

MN/CA IX was purified by a single cycle of adsorption–elution on sulphonamide-agarose, as described for other CAs (Falkbring et al, 1972). We used columns of p-aminomethylbenzene-sulphonamide-agarose (Sigma). Columns with adsorbed MN/CA IX were extensively washed with phosphate-buffered saline (PBS) sodium chloride (NaCl) 8.0 g $l^{-1}$, potassium chloride (KCl) 0.2 g $l^{-1}$, $KH_2PO_4$ 0.2 g $l^{-1}$, $Na_2HPO_4$ 1.15 g $l^{-1}$, pH = 7.2) and eluted with 0.1 mM acetazolamide (Sigma). All steps of purification were carried out at 0–5°C, pH 7.2, at physiological concentration of salts. Complete MN/CA IX+ was extracted with 1% Triton X-100 in PBS from Vero cells infected with vaccinia virus containing an insert of complete coding region of MN/CA IX as described (Závada et al, 1997). Before chromatography, the extract was diluted 1:6 with PBS and centrifuged for 1 h at 15 000 ***g***. Truncated MN/CA IX ΔTMΔIC was produced from an analogous construct except that the 3′ downstream primer for polymerase chain reaction (PCR) was: 5′-CGT CTA GAA GGA ATT CAG CTA GAC TGG CTC AGC A-3′. MN/CA IXΔ was shed into the medium, from which it was affinity purified after centrifugation as above. All steps of purification were monitored by dot-blots.

### Cells and media

The following cell lines were used: HeLa, CGL1 = non-tumorigenic hybrid HeLa × fibroblast, CGL3 = tumorigenic segregant from this hybrid. The origin of the cells and growth media were described previously (Závada et al, 1992, 1997). In addition, we used also HT29, a cell line derived from colorectal carcinoma (ATCC No. HBT-38).

### Cell adhesion test

The conditions of the test were described previously (Zavada et al, 1997). Briefly, 1 μg ml$^{-1}$ of purified MN/CA IX in 50 mM mono/bicarbonate buffer, pH 9.2, was adsorbed in 30-μl drops on the bottom of bacteriological 5-cm Petri dishes for 1.5 h. Then the drops were removed by aspiration and the dishes were 3 × rinsed with PBS and blocked with 50% fetal calf serum (FCS) in culture medium for 30 min. There were two variants of the test. In the first one (Figure 2), the whole bottom of the Petri dish was blocked with 50% of FCS, and the dishes were seeded with 5 ml of cell suspension ($10^5$ cells ml$^{-1}$). After overnight incubation, the cultures were rinsed with PBS, fixed and stained. In the other variant (Figure 4), only the area of adsorbed MN/CA IX was blocked and on top of MN/CA IX dots were added 30-μl drops of cell suspension in growth medium, containing added oligopeptides (or control without peptides). After incubation, rinsing and fixation, the cultures were stained with 0.5% Trypan blue in 50 mM Tris buffer pH 8.5 for 1 h, rinsed with water and dried. Stained areas of attached cells were extracted with 10% acetic acid, the extracts transferred to 96-well plates and absorbance was measured at 630 nm on a microplate reader.

### ELISA

Purified GST-MN (Závada et al, 1993) at concentration 10 ng ml$^{-1}$ in carbonate buffer pH 9.2 was adsorbed for 3 h in Maxisorb strips (NUNC). After washing and blocking (1 h) with 0.05% Tween-20 in PBS, 50 μl well$^{-1}$ of the antibody + antigen mixtures were added. Final dilution of mAb M75 ascites fluid was $10^{-6}$; concentration of the peptides varied according to their affinity for M75 so as to allow determination of 50% end point. These mixtures were adsorbed for 1.5 h, followed by washing with Tween–PBS. Bound antibody was detected by antimouse IgG conjugate with peroxidase (SwAM-Px, SEVAC, Prague), diluted 1:1000. In the colour reaction OPD (o-phenylenediamine dihydrochloride, Sigma) 1 mg ml$^{-1}$ in 0.1 M citrate buffer pH 5.0 was used. To this hydrogen peroxide ($H_2O_2$) was added to final concentration 0.03%. This system is balanced so as to allow assay for antigen competing for M75 (Figure 3) as well as for peptides binding to the epitope of immobilized GST-MN (Figure 4).

### Peptides

The peptides used in this study were prepared by the solid-phase method (Merrifield, 1995) using the Boc/Bzl strategy. The peptide acids were prepared on PAM-resin and peptide amides on MeBHA resin. Deprotection and splitting from the resin was done by liquid hydrogen fluoride. The peptides were purified by C18 reverse phase high-performance liquid chromatography (RP HPLC) and characterized by amino acid analysis and FAB MS spectroscopy.

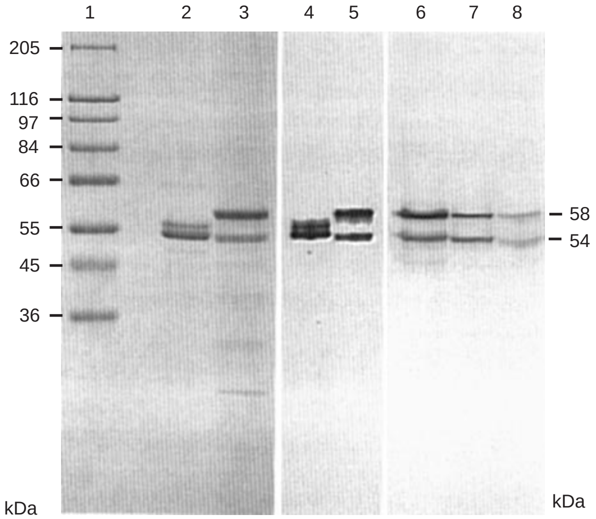

**Figure 1** PAGE and Western blot analysis of MN/CA IX protein. Lanes 1–3 = gel stained with Coomassie Blue; lanes 4–8 = blots developed with mAb M75 and immunoperoxidase. Lane 1 = $M_r$ reference proteins; lane 2 = purified MN/CA IX ΔTM, IC, 1.25 μg; lane 3 = purified MN/CA IX+, 2 μg; lane 4 = purified MN/CA IX ΔTM, IC, 0.125 μg; lane 5 = purified MN/CA IX+, 0.2 μg; lane 6 = extract from HT29 cells, 50 μl; lane 7 = extract from CGL3 cells, 50 μl; lane 8 = extract from HeLa cells, 50 μl

### Western blots

MN/CA IX antigens from polyacylamide gel electrophoresis (PAGE) gels were transferred to polyvinyl difluoride (PVDF) membranes (Immobilon P, Millipore) and developed with M75, followed by SwAM-Px (see above) and diaminobenzidine (Sigma) with $H_2O_2$. For dot-blots we used nitrocellulose membranes.

### Phage display

Ph.D.-7 Phage Display Peptide Library kit was used for screening as recommended by manufacturer (New England Biolabs). A 96-well plate was coated with peptide F (legend to Figure 3). Biopanning was carried out by incubating $2 \times 10^{11}$ phage with target coated plate for 1 h. Unbound phages were washed away with TBST (50 mM Tris–HCl pH 7.5, 150 mM NaCl, 0.1% Tween-20) and specifically bound phages were eluted with M75 antibody (2 μg in 100 μl of TBS per well). Eluted phage was amplified and used for additional binding and amplification cycles to enrich the pool in favour of binding sequence. After 5 rounds, individual clones were picked, amplified and sequenced using T7 sequencing kit (Pharmacia).

### RESULTS

### Affinity chromatography of MN/CA IX protein

For purification of MN/CA IX protein we decided to use affinity chromatography on sulphonamide-agarose column, described previously for other CAs (Falkbring et al, 1972). The advantages of this method are simplicity and the fact that the whole procedure is carried out under non-denaturing conditions. Vaccinia virus vector with an insert of the complete *MN/CA9* cDNA, or with truncated cDNA (lacking transmembrane and intracellular domains) was employed as a source of MN/CA IX protein.

*© 2000 Cancer Research Campaign* 

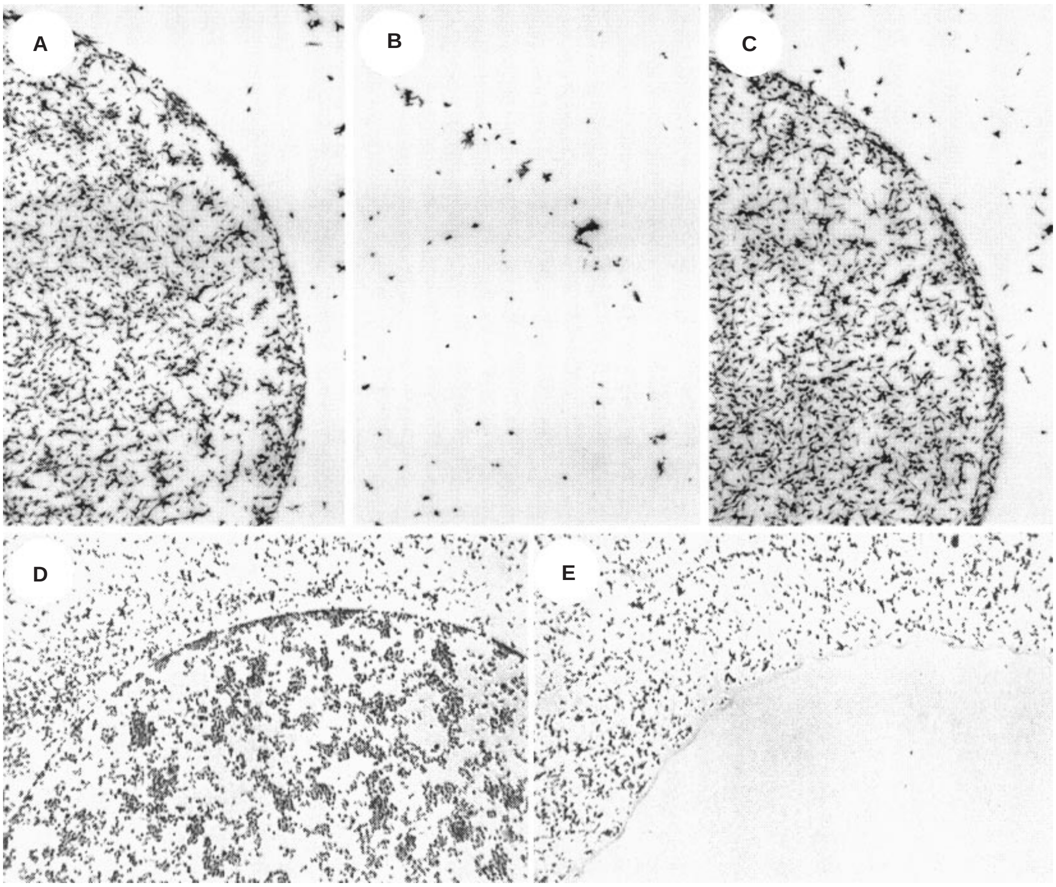

**Figure 2** Adhesion of cells to the dots of MN/CA IX protein immobilized in bacteriological Petri dishes. This plasticware (not treated for use with tissue cultures) allows no adhesion of CGL1 cells and a limited adhesion of HeLa. (**A**) CGL1 cells do attach and spread to the dot of adsorbed MN/CA IX. (**B**) Treatment of the dot with MAb M75 abrogates its binding capacity. (**C**) Treatment with MAb M16 (irrelevant for MN/CA IX) has no effect on cell attachment. (**D**) HeLa cells attach to complete MN/CA IX+. (**E**) HeLa cells do not attach to truncated MN/CA IXΔ

A single cycle of adsorption – elution yielded relatively pure proteins (Figure 1): MN/CA IX+ gave 2 bands of 54 and 58 kDa, MN/CA IXΔ of 54.5 and 56 kDa. These proteins strongly reacted with mAb M75 on Western blots. In extracts from HeLa, CGL3 and HT29 the blot revealed 2 bands of the same size as MN/CA IX+ purified from vaccinia virus construct.

### Adhesion of cells to MN/CA IX protein

MN/CA IX immobilized on hydrophobic plastic enabled attachment, spreading and growth of cells (Figure 2). Extremely low concentrations of MN/CA IX corresponding to 1 μg $ml^{-1}$ of purified protein in adsorption buffer were sufficient to cause this effect; other cell adhesion molecules are used in 10–50 × higher concentrations. Only complete MN/CA IX protein was active in cell adhesion test, truncated MN/CA IX did not support cell adhesion at all or it showed only a low adhesion activity and in some instances it even acted as a cell ‘repellent’.

Treatment of the dots of immobilized MN/CA IX with mAb M75 abrogated its capacity to attach the cells, but the control mAb M16, irrelevant for MN/CA IX had no effect. Blocking of cell attachment by M75 shows that the epitope is identical to or overlapping with the binding site of MN/CA IX for cell receptors.

### Identification of the epitope recognized by Mab M75

Preliminary mapping of M75 epitope employing partial sequences of extracellular parts of *MN/CA9* cDNA expressed from bacterial vectors and tested on Western blots located it in PG region. For exact mapping, our strategy was to synthesize partially overlapping oligopeptides of 15–25 aa covering the PG domain and test them in competition ELISA with M75. According to the results, this was followed by a series of 6–12 aa oligopeptides. A major part of the PG domain consists of a sixfold tandem repeat of six aa (aa 61–96); four repeats are identical (GEEDLP) and two contain two aa exchanged (SEEDSP and REEDPP) (Opavsky et al, 1996).

Figure 3 shows the result of competition ELISA with recombinant MN/CA IX and oligopetides synthesized according to partial sequences of the PG region. MN/CA IX+ and Δ produced in mammalian cells (proteins A and B) possessed a higher serological activity than any other protein or peptide included in this experiment; protein C–fusion protein GST-MN synthesized in bacteria was less active. Peptides D, E, F and G are spanning the PG region. Peptides D and F caused 50% inhibition at 1 ng $ml^{-1}$. These two oligopeptides are mutually non-overlapping, thus the epitope is repeated in both of them. Peptide E was 1000 × less active, probably due to a different conformation. Peptide G was inactive; thus it does not contain the M75 epitope.

*© 2000 Cancer Research Campaign*

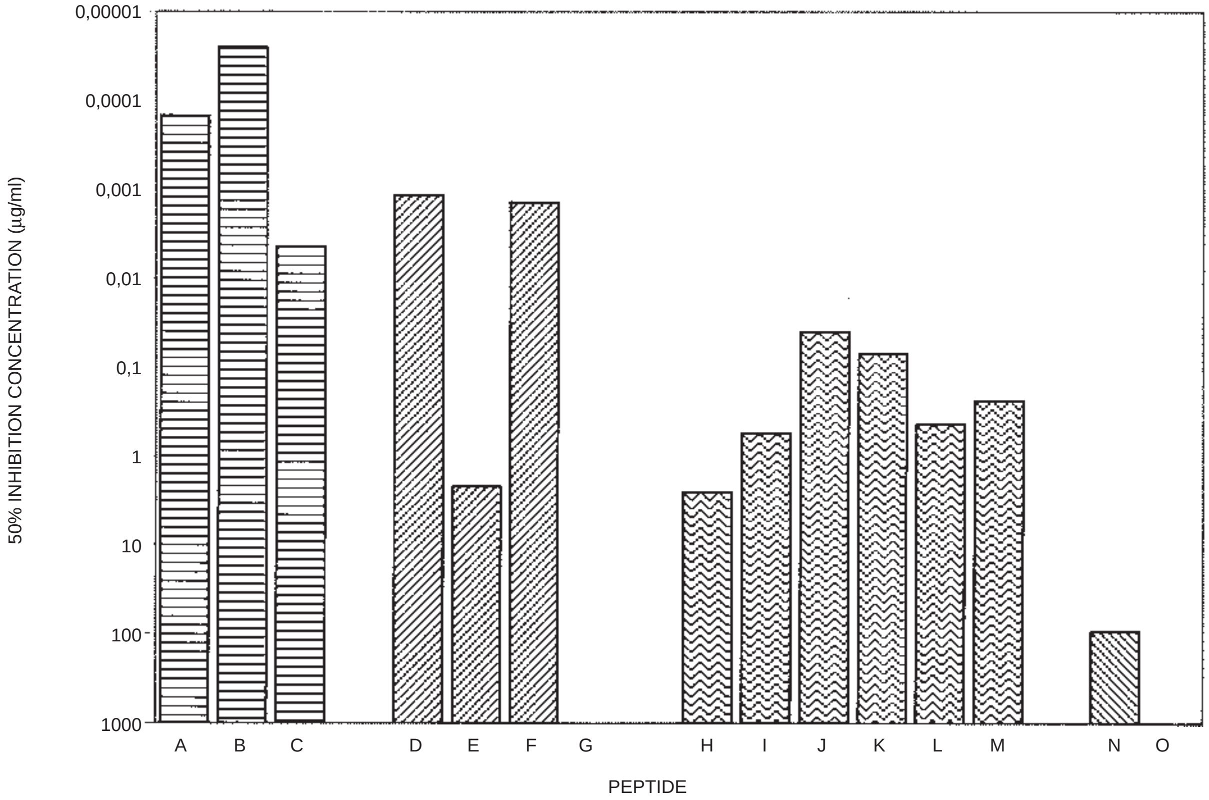

**Figure 3** Competition ELISA with proteins or oligopeptides related to MN/CA IX protein. Competing proteins/peptides: (**A–C**) Recombinant proteins. (**A**) MN/CA IX+; (**B**) MN/CA IXΔ; (**C**) GST-MN; (**D–G**) overlapping proteins covering the PG region: (**D**) GGSSGEDDPLGEEDLPSEEDSPC (aa 51–72); (**E**) GEEDLPSEEDSPREEDPPGEEDLPGEC (aa 61–85); (**F**) EDPPGEEDLPGEEDLPGEEDLPEVC (aa 75–98); (**G**) EVKPKSEEEGSLKLE (aa 97–111); (**H–M**) dodecapeptides: (**H**) GEEDLPGEEDLP; (**I**) EEDLPGEEDLPG; (**J**) EDLPGEEDLPGE; (**K**) DLPGEEDLPGEE; (**L**) LPGEEDLPGEED; (**M**) PGEEDLPGEEDL; (**N, O**) oligopeptides 7 + 2 aa: (**N**) APGEEDLPA; (**O**) AGEEDLPGA. Other oligopetides tested which proved negative in competition at 100 µg $ml^{-1}$ were oligopeptides 7 + 2 aa: AEEDLPGEA, AEDLPGEEA, ADLPGEEDA, ALPGEEDLA and all of the 6 + 2 aa permutations (AGEEDLPA, AEEDLPGA, AEDLPGEA, ADLPGEEA, ALPGEEDA and APGEEDLA). In oligopeptides D, E, F and G the C-terminal amino acid was present as acid, in all others as amide

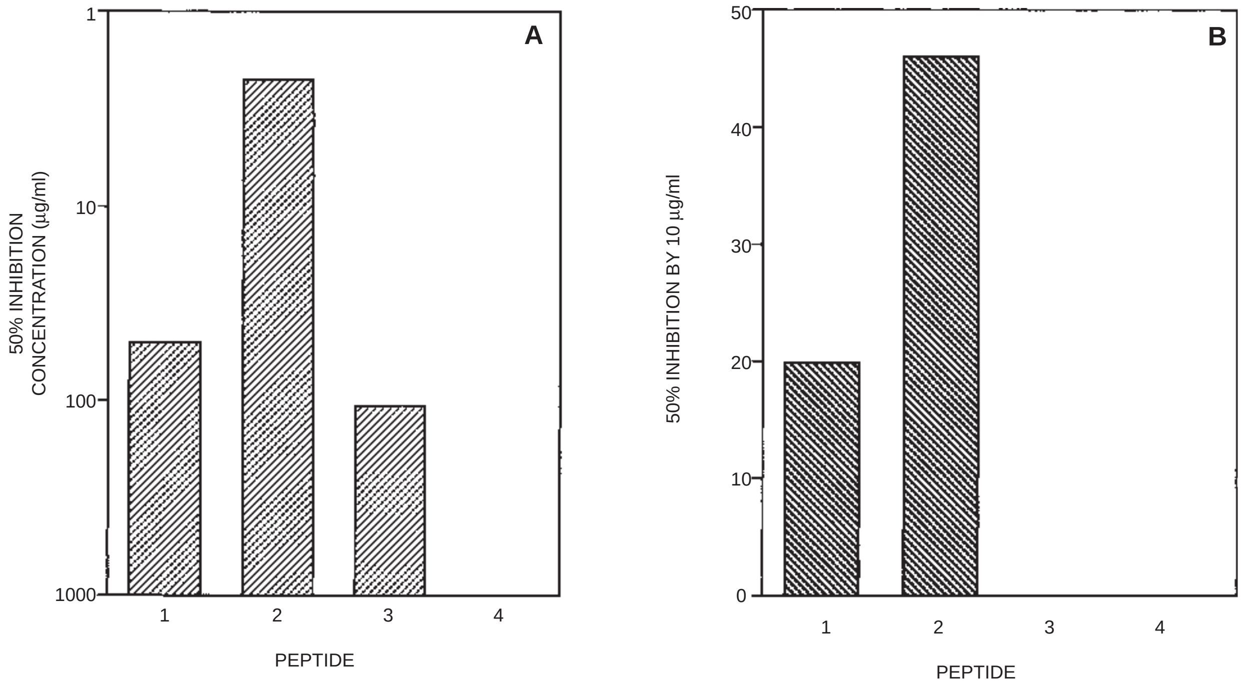

**Figure 4** Competition ELISA (**A**) and inhibition of cell attachment (**B**) by oligopeptides selected from phage display library. Peptides: 1 = AKKMKRRKA, 2 = AITFNAQYA, 3 = ASASAPVSA, 4 = AGQTRSPLA. In all 4 peptides the C-terminal alanine was present as amide

*© 2000 Cancer Research Campaign*

The next step for identifying the epitope was to synthesize oligopeptides containing all circular permutations of the motif GEEDLP repeated twice (peptides H–M), all six dodecapeptides were serologically active (two more and four less so). Following series of seven aa sequences, flanked by alanine on both ends finally showed that the minimum serologically active sequence is the oligopeptide N = APGEEDLPA. None of still shorter oligopeptides (6 + 2 aa) competed in ELISA for M75.

It is clear that the affinity between these oligopeptides and mAb M75 very strongly increases with the size of peptide molecule.

### Attempts to demonstrate adhesion of cells to immobilized oligopeptides

Our initial plan was to follow the pioneering work of Piersbacher and Ruoslahti (1984). They linked tested oligopeptides to adsorbed bovine serum albumin by cross-linking agent SPDP (N-succinimidyl 3[pyridylhydro] propionate). This is why we added onto the C-end of oligopeptides D, E and F (Figure 3) cysteine, which would enable oriented linking to adsorbed albumin. We demonstrated linking of the peptides directly in Petri dishes by immunoperoxidase staining with M75. Unfortunately, CGL1 or CGL3 cells adhered to control albumin treated with SPDP and blocked with ethanolamine (in place of oligopeptides) as strongly as to bovine serum albumin (BSA) dots with linked oligopeptides. We were unable to abrogate this non-specific adhesion. Oligopeptides D, E and F adsorb only very poorly to bacteriological Petri dishes, not allowing the cell adhesion test.

Alternatively, we tested inhibition of cell adhesion to MN/CA IX dots by oligopeptides added to the media together with the cell suspension, as described (Piersbacher and Ruoslahti, 1984). All peptides listed in the legend to Figure 3 were tested at concentrations 100 and 10 $\mu g\ ml^{-1}$. None of them inhibited reproducibly the adhesion of CGL1 cells.

### Oligopeptides with affinity to M75 epitope which inhibit cell adhesion to MN/CA IX

As an alternative to monoclonal antibodies, we set out to select oligopeptides exerting affinity to M75 epitope as well as to MN/CA IX receptor binding site from a phage display library of random heptapeptides – Ph.D.-7. Our aim was to select phages containing the desired heptapeptides by panning on immobilized peptide F (see legend to Figure 3) and subsequent elution with M75. Eluted phage was multiplied in appropriate bacteria and subjected to four more cycles of panning and elution. From the selected phage population, ten plaques were picked, amplified and the heptapeptide-coding region was sequenced. Only three heptapeptides were represented: these, after adding alanine on both sides, are nonapeptides Nos 1, 2 and 3 from Figure 4. The last heptapeptide, synthesized again with added terminal alanines as nonapeptide No. 4, was identified by panning on GST-MN and eluted with acetazolamide. This last peptide has affinity to the active site of MN/CA IX carbonic anhydrase. We synthesized these peptides of 7 + 2 aa and tested them in competition ELISA and in cell adhesion inhibition. Both tests yielded essentially consistent results: peptide No. 2 showed the highest activity, peptide No. 1 was less active, peptide No. 3 was marginally positive only in ELISA, and peptide No. 4 was inactive.

### DISCUSSION

Purification of transmembrane proteins like MN/CA IX often poses technical problems because they tend to form aggregates with other membrane proteins due to their hydrophobic TM segments. To avoid this, we engineered truncated MN/CA IX ΔICΔTM, which is secreted into the medium. Indeed, truncated MN/CA IX was obtained in higher purity than MN/CA IX+ (Figure 1). Unfortunately, this protein was of little use for our purposes, since it was inactive in the cell adhesion test (Figure 2). Such a situation has also been described for other cell adhesion molecules: their shed, shortened form either assumes an inactive conformation, or it adsorbs to hydrophobic plastic 'upside down', while complete proteins adsorb by hydrophobic TM segments in the 'correct' position.

MN/CA IX protein forms oligomers of 150 kDa, linked by disulphidic bonds (Pastorekova et al, 1992; Závada et al, 1993). It was not known whether these are homo- or hetero-oligomers. Figure 1 suggests that these are probably homo-oligomers, most likely trimers, since on the gel stained with Coomassie blue no additional bands of intensity comparable to two bands specific for MN/CA IX appeared. It is also unlikely that there could exist an additional protein co-migrating with one of the two major MN/CA IX bands, since the intensity of their staining on the gel and on Western blots is well comparable.

There can be no doubt on the specificity of cell attachment to purified MN/CA IX+. It is abrogated by specific mAb M75, at a dilution 1:1000 of ascites fluid. This is a correction to our previous report (Závada et al, 1997) in which we observed that MN/CA IX produced by vaccinia virus vector and fusion protein GST-MN support cell adhesion, but we did not realize that GST anchor itself contains another binding site, which is not blocked by M75.

mAb M75 reacts excellently with MN/CA IX under any circumstances – with native antigen on the surface of living cells, with denatured protein on Western blots and with antigen in paraffin sections of biopsies fixed with formaldehyde, suggesting that the epitope is small and contiguous. In competition ELISA (Figure 3) the smallest sequence reactive with M75 was 7 + 2 aa, but the affinity between M75 and tested peptides strongly depended on their molecular weight. Complete MN/CA IX was 1 000 000 × more active than the smallest serologically active peptide in terms of weight/volume concentration. In terms of molar concentration this difference would be 150 000 000 ×. Oligopeptides of intermediate size also showed intermediate activities. It remains to be elucidated whether such differences in activity are due to the conformation depending on the size of the molecule, or to the fact that complete MN/CA IX contains several copies of the epitope, but the smallest molecule only one.

Considering the possibility that the epitope is identical with the cell adhesion structure in MN/CA IX, we can understand why we failed to detect inhibition of cell adhesion by the oligopeptides. The binding site is just not as simple as the prototype peptide, RGD (Piersbacher and Ruoslahti, 1984).

Naturally, one can argue that the size of MN/CA IX is about the same as of immunoglobulin molecule, and that binding of M75 to its epitope may sterically hinder a different sequence of cell attachment site. This objection has been made unlikely by blocking of both M75 epitope and of cell binding site by nonapeptides 7 + 2 aa. This result (Figure 4) strongly suggests that the epitope and the binding site are indeed identical.

 © 2000 Cancer Research Campaign

MN/CA IX and its PG region in particular appears to be a potential target molecule for therapy for the following reasons: (i) it is exposed on the cell surface; (ii) it is present in high percentage of certain human carcinomas; (iii) it is normally expressed MN/CA IX in the mucosa of alimentary tract which is not accessible to circulating antibodies, in contrast with the tumours; (iv) it is not shed (or only minimally) into the body fluids; (v) the motif GEEDLP is repeated 18 × on the surface of every MN/CA IX molecule. Oligopeptide display libraries are being employed in the first steps to develop new drugs (Winter, 1996). Selected oligopeptides can serve as lead compounds for the computerized design of new molecules, with additional properties required from a drug (DeCamp et al, 1996).

### ACKNOWLEDGEMENTS

This research was supported by grant No. 301/99/0356 from Grant Agency of the Czech Republic, grant No. 2/4103/98 from Slovak Grant Agency, by Chiron-Bayer and by Volkswagen research grant No. I/71 392. The authors are grateful to Drs O Machoň, L Kutinová and Š Němečková for the construction of vaccinia virus vectors with *MN/CA9* inserts and to Prof R Weiss and to Dr Z Palková for their help in preparing this paper.

### REFERENCES

DeCamp D, Ogden R, Kuntz I and Craik CS (1996) Site-directed drug design. In: *Protein Engineering. Principles and Practice*, Cleland JL and Craik CS (eds), pp. 467–505. Wiley-Liss: New York

Falkbring SO, Göthe PO and Nyman PO (1972) Affinity chromatography of carbonic anhydrase. *FEBS Lett* **24**: 229–235

Liao SY, Brewer C, Závada J, Pastorek J, Pastorekova S, Manetta A, Berman ML, DiSaia PJ and Stanbridge EJ (1994) Identification of the MN antigen as a diagnostic biomarker of cervical intraepithelial squamous and glandular neoplasia and cervical carcinomas. *Am J Pathol* **145**: 598–609

Liao SY, Aurelio ON, Jan K, Závada J and Stanbridge EJ (1997) Identification of the MN/CA9 protein as a reliable diagnostic biomarker of clear cell carcinoma of the kidney. *Cancer Res* **57**: 2827–2831

Merrifield B (1995) Solid-phase peptide synthesis. In: *Peptides: Synthesis, Structures and Applications*, Gutte B (ed), pp. 93–169. Academic Press: San Diego

Oosterwijk E, Ruiter DJ, Hoedemaeker PJ, Pauwels EKJ, Jonas U, Zwartendijk J and Warnaar SO (1986) Monoclonal antibody G250 recognizes determinant present in renal cell carcinoma and absent from normal kidney. *Int J Cancer* **38**: 489–494

Opavsky R, Pastorekova S, Zelnik V, Gibadulinova A, Stanbridge EJ, Závada J, Kettmann R and Pastorek J (1996) Human MN/CA9 gene, a novel member of the carbonic anhydras family: structure and exon to protein domain relationship *Genomics* **33**: 480–487

Pastorek J, Pastorekova S, Callebaut I, Mornon JP, Zelnik V, Opavrsky R, Zatovicova M, Liao S, Portetelle D, Stanbridge EJ, Zavada J, Burny A and Kettmann R (1994) Cloning and characterization of MN, a human tumor-associated protein with a domain homologous to carbonic anhydrase and putative helix-loop DNA binding segment. *Oncogene* **9**: 2877–2888

Pastorekova S, Zavadova Z, Kostal M, Babusikova O and Zavada J (1992) A novel quasi-viral agent, MaTu, is a two-component system. *Virology* **187**: 620–626

Piersbacher MD and Ruoslahti E (1984) Variants of the cell recognition site of fibronectin that retain attachment-promoting activity. *Proc Natl Acad Sci USA* **81**: 5985–5988

Saarnio J, Parkkila S, Parkkila AK, Haukipuro K, Pastorekova S, Pastorek J, Kairaluoma MI and Karttunen TJ (1998*a*) Immunohistochemical study of colorectal tumors expression of a novel transmembrane carbonic anhydrase, MN/CA IX, with potential value as a marker of cell proliferation. *Am J Pathol* **153**: 279–285

Saarnio J, Parkkila S, Parkkila AK, Waheed A, Casey MC, Zhou XY, Pastorekova S, Pastorek J, Karttunen T, Haukipuro K, Kairaluoma MI and Sly WS (1998*b*) Immunochemistry of carbonic anhydrase isozyme IX (MN/CA IX) in human gut reveals polarized expression in the epithelial cells with the highest proliferative capacity. *J Histochem Cytochem* **46**: 497–504

Steffens MG, Boerman OC, Oosterwijk-Wakka JC, Oosterhof GON, Witjes JA, Koenders EB, Oyen WJ, Buijs WC, Debruyne FMJ, Corstens FHM and Oosterwijk E (1997) Targeting of renal cell carcinoma with Iodine-131-labeled chimeric monoclonal antibody G250. *J Clin Oncol* **15**: 1529–1539

Turner JR, Odze RD, Crum CP and Resnick MB (1997) MN antigen expression in normal, preneoplastic, and neoplastic esophagus: a clinicopathological study of a new cancer-associated biomarker. *Hum Pathol* **28**: 740–744

Uemura H, Kitagawa H, Hirao Y, Okajima E, Debruyne FMJ and Oosterwijk E (1997) Expression of tumor-associated antigen MN/G250 in urologic carcinoma: potential therapeutic target. *J Urol* **157**: 377

Vermylen P, Roufosse C, Burny A, Verhest A, Bosschaerts T, Pastorekova S, Ninane V and Sculier J-P (1999) Carbonic anhydrase IX antigen differentiates between preneoplastic malignant lesions in non-small cell lung carcinoma. *Eur Respir J* **14**: 806–811

Winter J (1996) Bacteriophage display libraries. In: *Protein Engineering. Principles and Practice*, Cleland JL and Craik CS (eds), pp. 349–369. Wiley-Liss: New York

Závada J, Zavadova Z, Pastorekova S, Ciampor F, Pastorek J and Zelnik V (1993) Expression of MaTu-MN protein in human tumor cultures and in clinical specimens. *Int J Cancer* **54**: 268–274

Závada J, Zavadova Z, Machon O, Kutinova L, Nemeckova S, Opavsky R and Pastorek J (1997) Transient transformation of mammalian cells by MN protein, a tumor-associated cell adhesion molecule with carbonic anhydrase activity. *Int J Oncol* **10**: 857–863

*© 2000 Cancer Research Campaign*
